# Dietary salt intake worsens the Th17-dependent inflammatory profile of patients with cirrhosis

**DOI:** 10.1172/jci.insight.191354

**Published:** 2025-07-24

**Authors:** Amalia Tzoumpa, Beatriz Lozano-Ruiz, Yin Huang, Joanna Picó, Alba Moratalla, María Teresa Pomares, Iván Herrera, Juanjo Lozano, María Rodríguez, Cayetano Miralles, Pablo Bellot, Paula Piñero, Fabián Tarín, Pedro Zapater, Sonia Pascual, José Manuel González-Navajas

**Affiliations:** 1 Alicante Institute for Health and Biomedical Research (ISABIAL), Hospital General Universitario Dr. Balmis, Alicante, Spain.; 2 IDiBE Institute, Miguel Hernández University of Elche (UMH), Elche, Spain.; 3 Networked Biomedical Research Center for Hepatic and Digestive Diseases (CIBERehd), Instituto de Salud Carlos III, Madrid, Spain.; 4 Liver Unit, and; 5 Haematology Service, Hospital General Universitario Dr. Balmis, Alicante, Spain.; 6 Department of Pharmacology, Paediatrics and Organic Chemistry, Miguel Hernández University of Elche (UMH), Elche, Spain.

**Keywords:** Hepatology, Immunology, Inflammation, Fibrosis, Hepatitis, T cells

## Abstract

**BACKGROUND:**

Liver cirrhosis is characterized by chronic inflammation and fibrosis, with Th17 cells playing a crucial role in its progression. Recent evidence suggests that dietary salt influences immune diseases by modulating Th17 differentiation. This study assessed the impact of dietary salt on Th17-driven inflammation in patients with compensated cirrhosis and explored its effects on liver injury in mouse models.

**METHODS:**

A nondrug, open-label, nonrandomized study involved 37 patients with compensated cirrhosis, who were given personalized guidelines to reduce salt intake over 3 months. Changes in Th17-driven inflammation and liver function markers were assessed at baseline and after salt restriction. In parallel, the impact of a high-salt diet on hepatic CD4^+^ T cells was analyzed in mouse models of acute liver injury and fibrosis.

**RESULTS:**

High salt intake was associated with Th17-mediated inflammation and correlated with markers of impaired liver function in these patients. Importantly, moderating salt intake through a personalized nutritional intervention was sufficient to reduce CD4^+^ T cell–mediated inflammation. Furthermore, analysis of RNA-seq data revealed enrichment of salt-induced Th17 gene signatures in both liver tissue and peripheral cells from patients with liver disease. Similarly, mice fed a high salt diet showed hepatic enrichment of Th17 cells and exacerbated liver fibrosis upon injury. Mechanistic studies revealed that high sodium conditions activated NF-κB and induced IL-6 production in hepatocytes, which may promote Th17 responses.

**CONCLUSION:**

Dietary salt exacerbates Th17-driven inflammation and contributes to cirrhosis progression. Salt reduction may represent a viable therapeutic approach to manage inflammation in compensated cirrhosis.

**FUNDING:**

Grants PI19/01554 and PI22/01907 from Instituto de Salud Carlos III (Madrid, Spain), CDEI-03/20-A and CIPROM/2023/4 from Generalitat Valenciana (Valencia, Spain), CNS2023-145676 from the National Research Agency (AEI) (Madrid, Spain), and LCF/BQ/D121/11860047 from La Caixa Foundation (Barcelona, Spain).

## Abstract 2

In this manuscript, we show that dietary salt intake may contribute to the progression of compensated liver cirrhosis by modulating Th17-dependent inflammation.

## Introduction

Liver cirrhosis is a widespread condition responsible for approximately 1 million deaths annually, ranking as the 11th leading cause of death globally (1). Various risk factors such as obesity, steatotic liver disease, excessive alcohol intake, hepatitis B or C infections, and autoimmune disorders can trigger persistent inflammation leading to fibrosis and eventual cirrhosis (2).

T helper 17 cells (Th17) are proinflammatory cells that produce cytokines of the IL-17 family (3). They contribute to inflammation and fibrosis in chronic liver diseases by promoting the expression of inflammatory and profibrotic cytokines (4, 5). Elevated levels of IL-17A are found in the serum of patients with alcohol-related liver disease, chronic hepatitis B, and chronic hepatitis C, and correlate with increased numbers of circulating Th17 cells and fibrosis (6, 7). In addition, hepatic Th17 cells have been associated with poor prognosis in patients with hepatocellular carcinoma (HCC) (8). Finally, several studies have described the pathogenicity of Th17 cells in the progression of metabolic dysfunction–associated steatotic liver disease (MASLD) (9–11).

Dietary salt (sodium chloride; NaCl) affects immune activation and immune-mediated diseases (12–14). Elevated Na^+^ enhances Th17 cell differentiation in vitro and accelerates autoimmune diseases in vivo. Mechanistically, NaCl activates NFAT5 and SGK1, which, in turn, regulates IL-23R expression and stabilizes the Th17 phenotype (15, 16). On the other hand, high NaCl concentrations have been shown to impair Treg function through the upregulation of IFN-γ in Treg cells and downregulation of IL-10 via SGK1 and metabolic perturbations (17–19).

Given the critical role of inflammation in the progression of liver cirrhosis, it is crucial to understand how salt intake influences immune responses in patients with compensated cirrhosis. Therefore, this study aimed to investigate the effects of salt intake on CD4-mediated inflammation and its potential impact on liver function markers in these patients. We also assessed whether a 3-month salt restriction protocol could positively impact these inflammatory and liver function parameters. Our findings indicate that dietary salt may contribute to the progression of liver cirrhosis by modulating Th17-dependent inflammatory responses, suggesting that reducing salt intake could be a possible therapeutic strategy for managing inflammation in these patients.

## Results

### Clinicopathological characteristics of patients.

**Figure 1:**
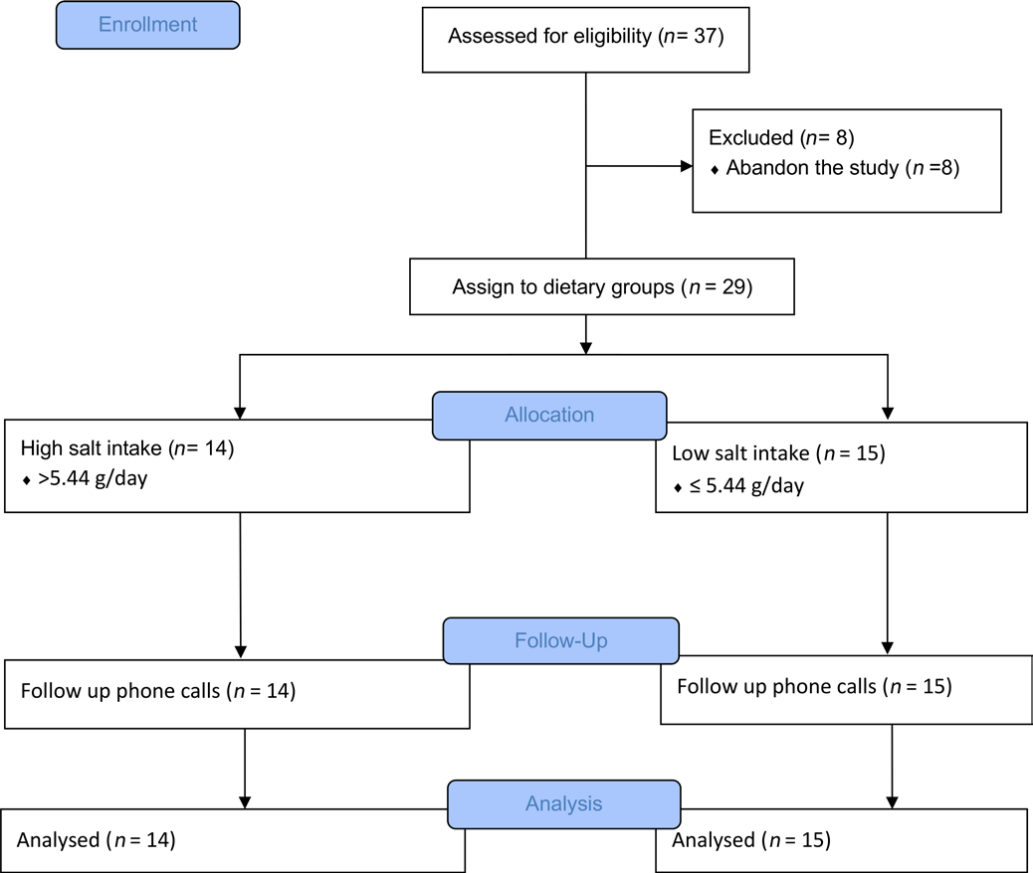
CONSORT study flow diagram. Flow chart illustrates the number of patients that were included in the study, allocated to each dietary group and analyzed. Out of the 37 patients that were initially recruited, 8 withdrew from the study due to unwillingness to adhere to the dietary recommendations. All patients were classified as Child-Pugh A.

**Figure 2:**
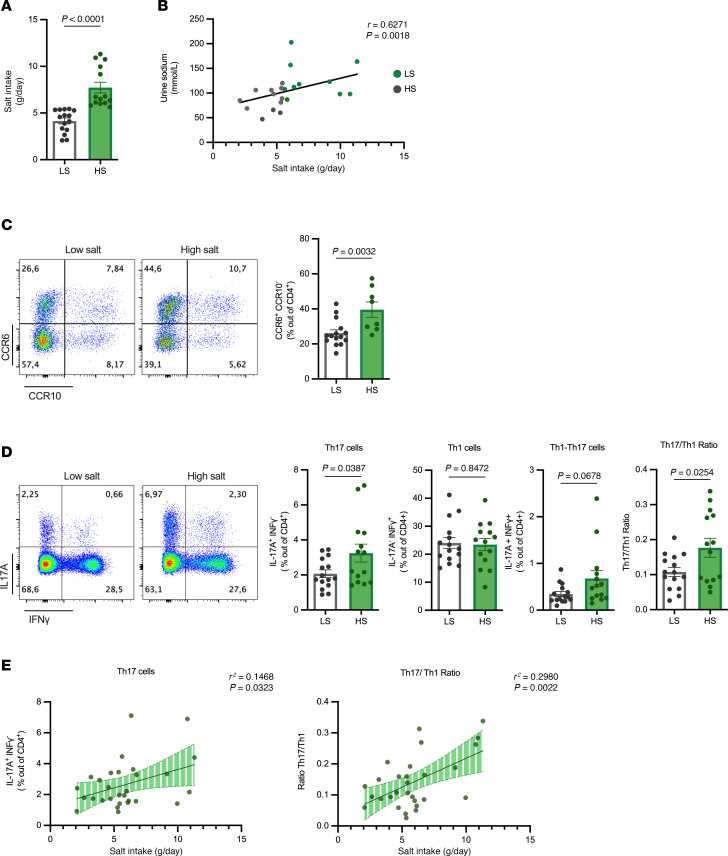
Baseline high salt intake values correlate with increased circulating Th17 cells in patients with compensated cirrhosis. Patients with compensated cirrhosis were categorized based on their baseline daily salt intake into 2 groups, referred to as ‘low salt’ (LS, *n =* 14) and ‘high salt’ (HS, *n =* 15), and their Th17 profile was analyzed in peripheral blood mononuclear cells (PBMCs) by flow cytometry. (**A**) Quantification plots of average daily salt intake in both groups of patients. (**B**) Spearman correlation scatterplot showing the association between 24-hour urine sodium and daily salt intake in both groups of patients (*n =* 22). (**C**) Representative flow cytometry plots (left) and quantification bar graphs (right) of CD4^+^CCR6^+^CCR10^–^ cells in PBMCs. (**D**) Representative flow cytometry plots (left) and quantification bar graphs of Th17 cells (IL-17Α^+^ IFNγ^–^), Th1 cells (IL-17Α^–^ IFNγ^+^), Th17-Th1 cells (IL-17Α^+^ IFNγ^+^), and the ratio of Th17 to Th1 cells. (**E**) Linear regression analysis showing the association between salt intake and circulating Th17 cells (left) or the Th17 to Th1 ratio. Data in **A**, **C**, and **D** were analyzed using the Mann-Whitney U test and unpaired *t* test correspondently and are displayed as mean ± SEM. Spearman correlation was used for data in **B** and simple linear regression with 95% CIs was shown in **E**.

Out of the 37 patients with compensated cirrhosis that were initially recruited, all of whom were classified as Child-Pugh A, 8 patients abandoned the study and were therefore excluded from the analysis (Figure 1). Daily salt intake for the remaining 29 patients was estimated using food questionnaires and individual interviews. Then, based on the median intake value of this cohort (5.44 g/day), the patients were categorized into two groups: “low salt” (LS, *n =* 15) and “high salt” (HS, *n =* 14) intake. The LS and HS groups had an average daily salt intake of 4.15 g/day and 7.72 g/day, respectively (Figure 2A). First, we compared clinical characteristics between the 2 groups at baseline (Table 1). The median age was significantly lower in the HS group (*P* < 0.001). No other differences were observed in clinical variables, including systolic and diastolic blood pressure and several markers of liver damage and function such as AST, ALT, GGT, total bilirubin, albumin, INR, and platelets, indicating that patients in both groups were at comparable clinical stages (Table 1). As expected, 24-hour urinary sodium excretion was elevated in the HS group (Table 1 and Supplemental Figure 1A; supplemental material available online with this article; https://doi.org/10.1172/jci.insight.191354DS1), and there was a positive correlation with the daily intake of salt calculated through food questionnaires (Figure 2B), which supports the validity of these calculations (Figure 2B).

### High salt intake boosts Th17 cells in compensated cirrhosis patients.

Dietary salt intake enhances Th17-mediated responses in healthy individuals (20, 21). To investigate whether salt intake could affect the systemic Th17-mediated response in cirrhotic patients, we analyzed the frequency of CD4^+^CCR6^+^CCR10^–^ cells, which are classical surface markers of Th17-like cells in PBMCs (22). We found a significant increase in CCR6 expression within the HS group compared with the LS group (Figure 2C), along with a significant positive correlation with salt intake (Supplemental Figure 2B). Similar results were found in CD3^+^CD4^–^ T cells (Supplemental Figure 1, C and D), although the expression of CCR6 was notably lower in this population. These results indicate that high salt intake boosts circulating CD4^+^CCR6^+^CCR10^–^ cells in patients with cirrhosis. Next, to further characterize the Th17 population, we isolated CD4^+^ T cells and analyzed the production of IL-17A and IFN-γ by intracellular flow cytometry after in vitro stimulation. Of note, the frequency of IL-17A–producing Th17 cells correlated with the frequency of circulating CD4^+^CCR6^+^CCR10^–^Th17-like cells (Supplemental Figure 1E). More importantly, we detected a significant increase in IL-17A^+^ cells within the HS group, accompanied by a not-statistically significant increase in IFNγ^+^IL-17A^+^ double-positive cells (Th1-Th17 subset) (Figure 2D). Moreover, the ratio of Th17 to Th1 cells was further augmented, indicative of a pronounced shift towards Th17 cell–mediated immunity in patients with cirrhosis and increased dietary salt intake. Conversely, the level of Th1 cells remained unchanged regardless of salt intake (Figure 2D). Lastly, we found moderate positive correlations between daily salt intake and Th17 cells, Th1-Th17 cells, and the Th17-to-Th1 ratio, while no significant correlation was found with Th1 cells (Figure 2E and Supplemental Figure 1F).

Similar findings were obtained when we analyzed supernatants from purified CD4^+^ T cells (99% purity; Supplemental Figure 2A) that were stimulated with anti-CD3 and anti-CD28 antibodies for 24 hours. First, CD4^+^ T cells from the HS group produced higher amounts of IL-17A while the secretion of IFN-γ or IL-10 remained unaltered (Supplemental Figure 2B). We also found moderate positive correlations between salt intake and both the in vitro production of IL-17A and the IL-17A to IFN-γ ratio (Supplemental Figure 2C). Notably, there was also a moderate association between salt intake and the IL-17A–to–IL-10 production ratio (Supplemental Figure 2, B and C), which could be of prognostic relevance in liver cirrhosis according to recent studies (23, 24). Last, to further characterize the effect of salt on CD4^+^ T cell functionality, we measured the production of IL-6, TNF, IL-2, IL-4, and GM-CSF; however, we did not observe any significant correlation between these cytokines and salt intake (Supplemental Figure 2D). Taken together, these data demonstrate that higher salt intake in patients with compensated cirrhosis specifically promotes Th17-mediated responses, shifting the balance of CD4^+^ helper subsets towards Th17 inflammation. Moreover, our data suggest that this alteration is likely caused by an increase in Th17 cells rather than a decrease in Th1 or Treg cells or their ability to produce IFN-γ or IL-10.

### Clustering analysis links the salt/Th17 axis with a phenotype of impaired liver function.

**Figure 3:**
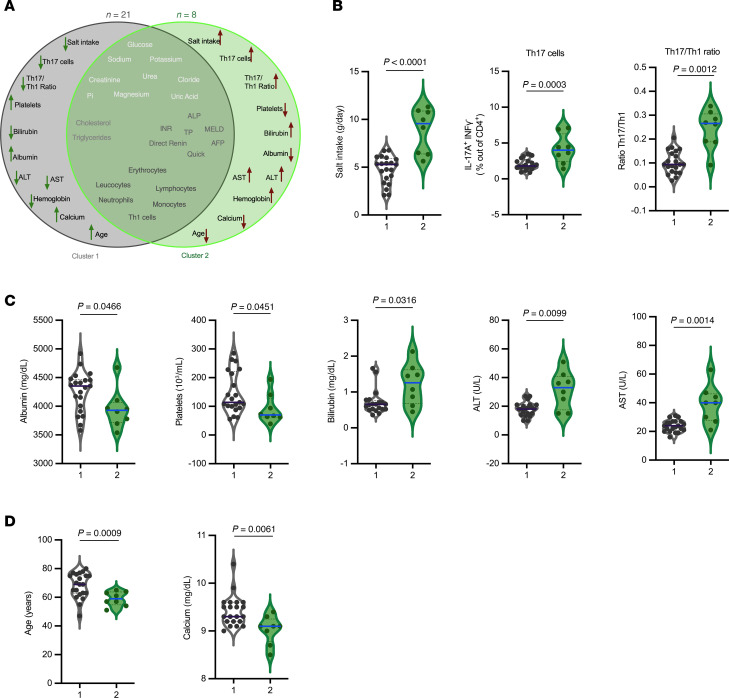
Unsupervised clustering of clinical and immunological variables links the Salt/Th17 axis with markers of impaired liver function in cirrhotic patients. (**A**) Unsupervised hierarchical clustering analysis was performed using all recorded quantitative clinical variables and immunological parameters (Th17%, Th1%, Th17 to Th1 ratio), identifying 2 patient clusters (cluster 1, *n =* 21; cluster 2, *n =* 8). (**B**–**D**) Violin plots showing the distribution of the indicated variables across clusters. Data were analyzed using the Mann-Whitney U test.

To investigate whether the effect of salt intake on Th17 cells could affect the clinical status of the patients, we performed an unsupervised hierarchical clustering analysis. This analysis incorporated all quantitative clinical variables recorded from the patients, which are detailed in Supplemental Table 1, as well as the experimental variables: percentage of Th17 and Th1 cells, and the Th17 to Th1 ratio. This analysis categorized patients into 2 distinct clusters: 21 patients were grouped into cluster 1, and 8 patients into cluster 2 (Figure 3A). Patients in cluster 2 showed markedly elevated salt consumption and a greater frequency of Th17 cells (Figure 3B). Furthermore, they exhibited characteristics of impaired liver function, such as reduced levels of albumin and platelets, along with significantly elevated levels of bilirubin, ALT, and AST (Figure 3C). No significant variance was observed in the remaining biological parameters between the 2 groups, apart from age and a minor change in serum calcium (9.3 vs 9.1 mg/dL, *p* = 0.006) (Supplemental Table 1 and Figure 3D). This clustering approach allowed us to identify distinct patient profiles, highlighting the potential association between high salt intake, Th17 response, and impaired liver function. The findings suggest a specific correlation between salt-induced Th17 cells and markers of liver function in cirrhotic patients, independently of other variables, and imply that salt intake might exacerbate the severity of liver disease by enhancing Th17-dependent inflammation.

### Sodium-induced and pathogenic Th17 gene signatures are enriched in liver disease.

**Figure 4:**
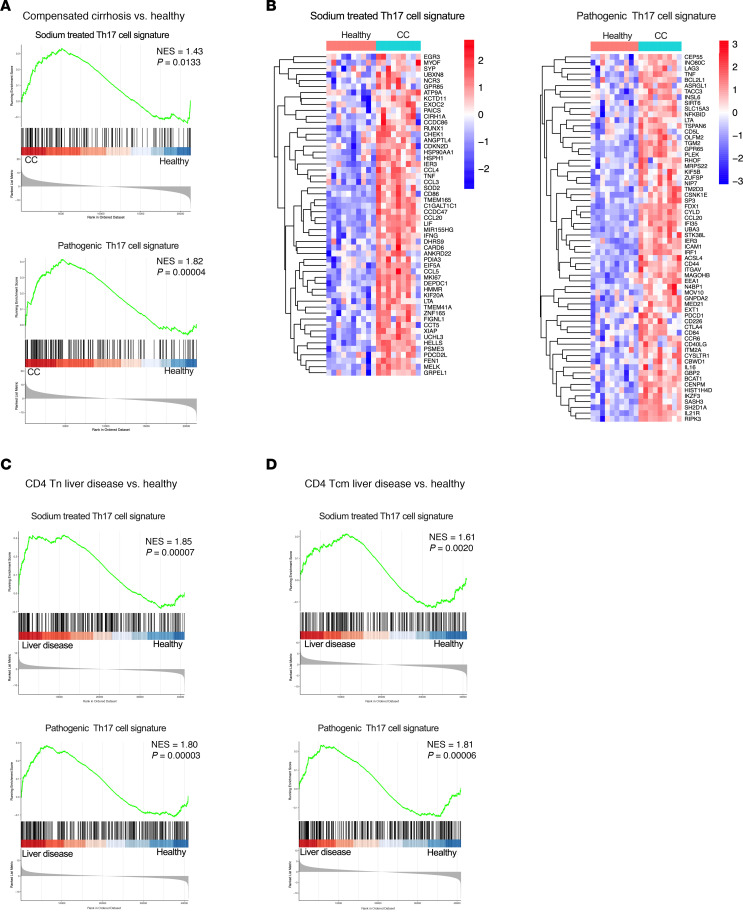
RNA-seq analysis reveals enrichment of Th17-related transcriptional signatures in liver disease. (**A**) GSEA of bulk RNA-seq data from liver tissue of patients with compensated cirrhosis (CC) (*n =* 9) and nondiseased controls (*n =* 11). (**B**) Heatmaps showing the expression of the top upregulated genes from the sodium-induced Th17 signature (left) and pathogenic Th17 signature (right) in CC liver samples. (**C** and **D**) GSEA plots of bulk RNA-seq data from peripheral naive (**C**) or central memory (**D**) CD4^+^ T cells of patients with liver disease (*n =* 9) and healthy individuals (*n =* 8).

We next performed a metaanalysis on published bulk RNA-seq datasets from liver samples of patients with compensated cirrhosis as well as from fragments of livers from humans without disease. We first conducted GSEA and heatmap analyses to interrogate a gene set known to be upregulated in sodium-induced Th17 cells in vitro (15). The analysis revealed a significant upregulation of this signature gene set in compensated cirrhosis patients compared with healthy samples, with 65 enriched genes (Figure 4, A and B). Subsequently, using the same dataset, GSEA and heatmap analyses were carried out for a Th17 pathogenic signature gene set previously identified through single-cell RNA-seq (25). These analyses also evidenced a substantial enrichment of this signature in patients with compensated cirrhosis (Figure 4, A and B), suggesting the involvement of pathogenic Th17 cells in this disease. Furthermore, to interrogate the potential systemic role of Th17 cells, we performed a GSEA analysis on published bulk RNA-seq data from CD4^+^ T cells obtained from peripheral blood of patients with liver disease and healthy individuals. The analysis revealed a high enrichment of the sodium-induced Th17 gene signature in both naive CD4^+^ T cells (Figure 4C) and central memory CD4^+^ T cells (Figure 4D), with less pronounced enrichment in the effector memory subset (Supplemental Figure 3A). Similarly, the GSEA analysis for the pathogenic Th17 signature indicated enrichment in naive and central memory CD4^+^ T cells (Figure 4, C and D). Overall, these findings demonstrate that Th17 cell signatures, both sodium-induced and pathogenic, are upregulated in liver tissue and peripheral blood of patients with cirrhosis. This suggests a prominent role for sodium-induced Th17 cells in the pathogenesis of this disease.

### Dietary salt restriction affected the Th-mediated immune response in patients with cirrhosis.

**Figure 5:**
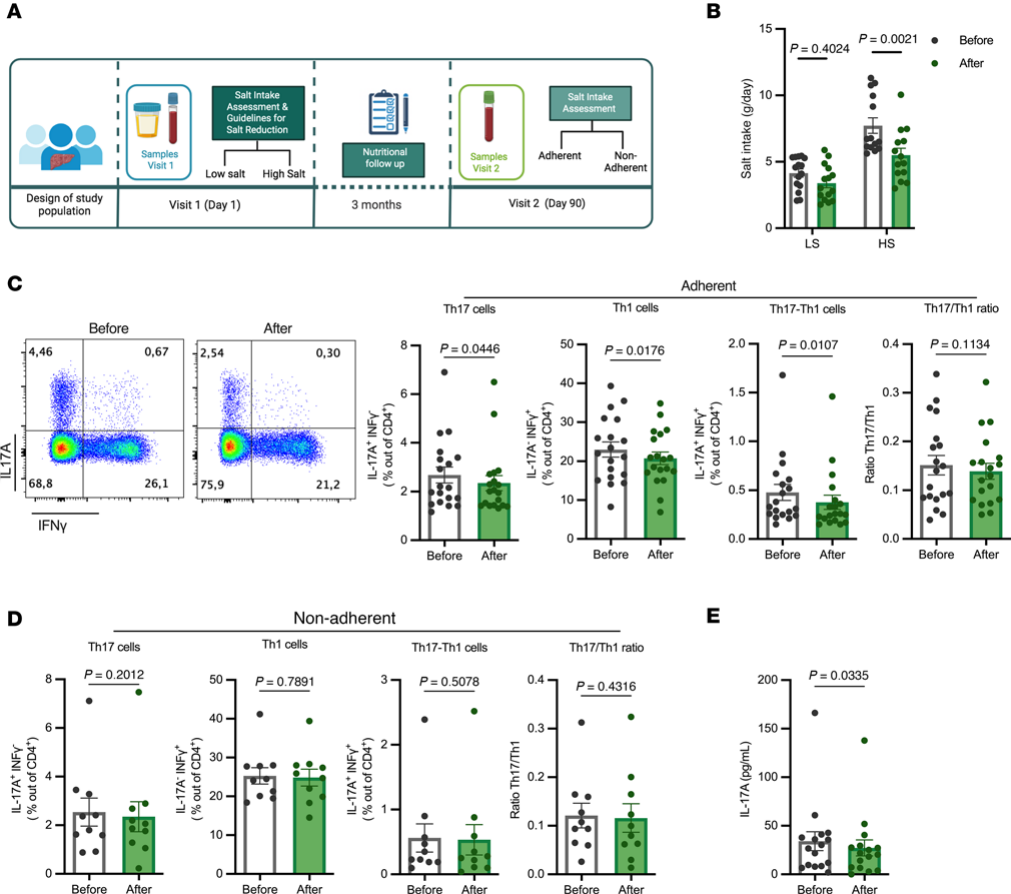
Dietary salt restriction decreases systemic Th17 responses in patients with cirrhosis. (**A**) Schematic representation of the dietary intervention study. All patients (*n =* 29) received personalized nutritional guidelines to reduce salt intake during a total of 3 months, without altering total calorie intake or other nutrients. (**B**) Paired comparison of estimated salt intake before and after salt restriction. (**C** and **D**) Representative flow cytometry plots (left) and quantification graphs of Th17 cells (IL-17Α^+^ IFNγ^–^), Th1 cells (IL-17Α^–^ IFNγ^+^), Th17-Th1 cells (IL-17Α^+^ IFNγ^+^), and the Th17 to Th1 ratio in adherent (**C**) and nonadherent (**D**) groups, before and after the intervention. (**E**) Paired comparison of IL-17Α production by peripheral CD4^+^ T cells after 24-hour in vitro stimulation (*n =* 25). Data are presented as mean ± SEM and analyzed using 2 way ANOVA, multiple comparison for **B** and the Wilcoxon matched-pairs test for **C**–**E**.

Based on the positive association between high salt intake and markers of systemic Th17 activation and impaired liver function outlined in this study (Figures 2–4), as well as the established role of the Th17/IL-17Α axis in exacerbating liver pathologies, we reasoned that reducing dietary salt intake may have a positive effect in the Th17-mediated inflammatory profile during compensated cirrhosis. Hence, we conducted a dietary intervention study in which all patients received personalized nutritional guidelines to reduce salt intake without affecting other nutrients or total calorie intake (Figure 5A). Patients then were asked to record food intake for a period of 3 months, during which they received follow-up calls as described in the methods. After 3 months, the patients were interviewed again to calculate salt intake and to collect a new blood sample (Figure 5A). As anticipated, there was a significant reduction in the average daily salt intake across the entire patient cohort after the implementation of the dietary guidelines (Figure 5B), while total calorie intake remained unchanged (Supplemental Figure 4A). However, adherence to the salt restriction guidelines varied among participants. Consequently, patients were categorized into adherent (*n =* 20) and nonadherent (*n =* 9) groups based on whether they achieved an average daily salt reduction of > 0.5 g/day or ≤ 0.5 g/day, respectively. Importantly, the adherent group showed a statistically significant decrease in IL-17A^+^IFN-γ^–^ Th17 cells and the IL-17A^+^IFN-γ^+^ double-positive Th1-Th17 cells after dietary salt restriction (Figure 4C). Interestingly, the low salt diet also resulted in a reduction of the IL-17A^–^IFNg^+^ Th1 population (Figure 5C). As a consequence, although the Th17-to-Th1 ratio was also slightly decreased, the change was not statistically significant (Figure 5C). In contrast, the nonadherent group of patients did not exhibit a significant change in any of the Th populations or in the Th17-to-Th1 ratio at the end of the study (Figure 5D). Moreover, we purified and stimulated CD4^+^ T cells in vitro to analyze cytokine production in response to TCR stimulation. As expected, in vitro IL-17A production decreased after implementing the salt restriction guidelines in the adherent group (Figure 5E). Importantly, no differences were observed in the production of other cytokines (Supplemental Figure 4B), suggesting that modulating salt intake has a specific impact on the Th17 phenotype in these patients. Regarding the clinical variables, we did not observe any significant effects from the salt restriction protocol within the study’s timeframe (Supplemental Figure 5). Together, these data suggest that even a short period of moderate salt restriction (3 months) may modulate systemic Th17 responses in patients with compensated cirrhosis.

### High salt diet exacerbates liver injury and fibrosis and modulates immune responses in mouse models.

We next aimed to test whether high salt diet (HSD) could affect hepatic Th17-dependent responses in vivo in an acute liver injury model. C57BL/6J mice were fed either a normal-diet (ND) or HSD for 3 weeks, followed by diethylnitrosamine (DEN) or vehicle treatment. Mice were sacrificed 48 hours after DEN administration. While HSD alone did not cause liver damage, it exacerbated DEN-induced injury, as shown by increased necrotic areas (Supplemental Figure 6A). Moreover, IHC revealed more IL-17A^+^ cells in HSD-fed mice after DEN (Supplemental Figure 6B), and CD4^+^ cells from these livers showed higher ROR-γ mRNA expression, a key factor in Th17 differentiation (Supplemental Figure 6C).

**Figure 6:**
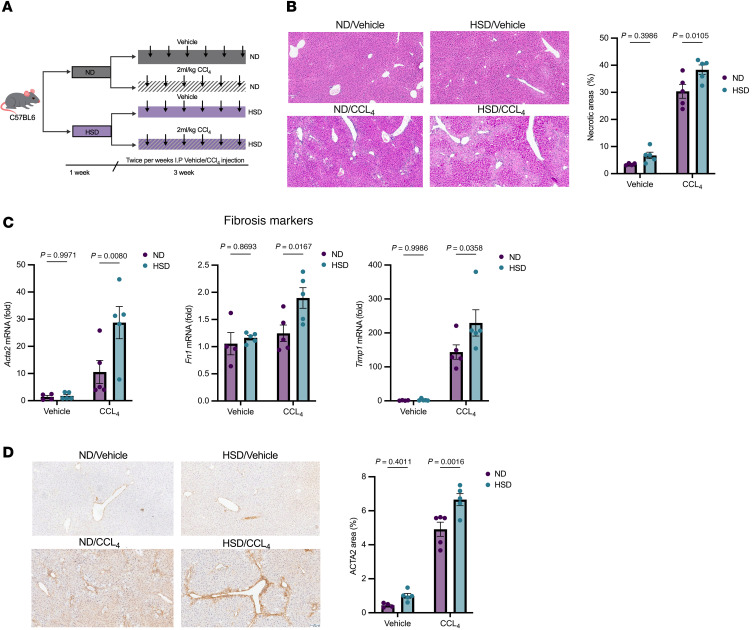
High salt diet exacerbates liver fibrosis in a mouse model of CCl_4_-induced liver injury. (**A**) Schematic representation of the experimental design. C57BL/6J mice were fed either normal diet (ND) or high salt diet (HSD) for 1 week and were then additionally subjected to carbon tetrachloride (CCl_4_) or vehicle injections (2 mL/kg) twice weekly for 3 weeks to induce liver fibrosis. (**B**) Representative images of H&E-stained liver sections (left) and quantification of the necrotic areas in each group of mice (right). Original magnification, x200. (**C**) Hepatic mRNA expression of fibrosis markers (*Acta2, Fn1, Timp1*). (**D**) ACTA2 protein levels assessed by IHC. Representative images (left) and quantification plots (right). Original magnification, x200. Data were analyzed using the 2way-ANOVA multiple comparisons test (**B**–**D**) and are displayed as mean ± SEM (ND/Vehicle *n =* 4, HSD/Vehicle *n =* 5, ND/CCl_4_ *n =* 5, HSD/CCl_4_ *n =* 5). Data were generated in a single experiment.

Furthermore, we investigated the impact of HSD on liver fibrosis and its associated immune cell dynamics in the carbon tetrachloride (CCl_4_) model. C57BL/6J mice were fed either ND or HSD for one week and were then additionally subjected to CCl_4_ or vehicle injections (2 mL/kg) twice weekly for 3 weeks to induce liver fibrosis (Figure 6A). As in the DEN model, HSD alone did not result in liver damage or infiltration of immune cells (Figure 6B). However, it exacerbated CCl_4_-induced injury, as evidenced by increased necrotic areas (Figure 6B). Mice on HSD exhibited significantly elevated mRNA levels of fibrosis markers such as *Acta2*, *Fn1*, and *Timp1* upon CCl_4_ treatment compared to ND groups, indicating that HSD augments the transcriptional profibrotic response in the context of CCl_4_-induced liver injury. No difference was found between the vehicle-treated groups, suggesting that short-term HSD alone is not enough to induce a profibrotic response (Figure 6C). Similarly, IHC staining of ACTA2 (Actin Alpha 2, Smooth Muscle) further indicated enhanced fibrosis in the liver of mice fed HSD (Figure 6D).

**Figure 7:**
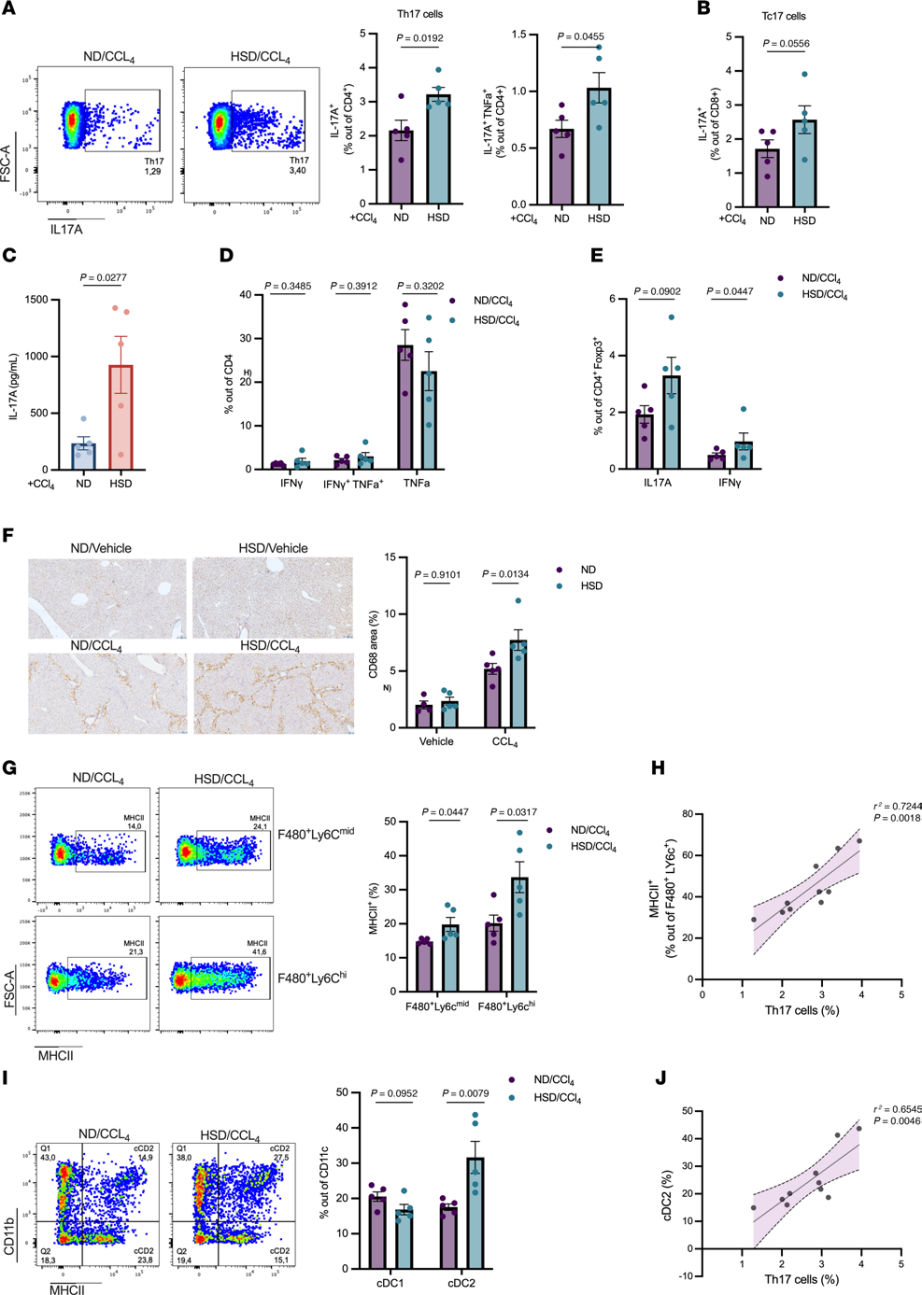
High salt diet modulates hepatic immune responses in CCl4-induced fibrosis. (**A**) Representative flow cytometry plots (left) and quantification graphs of CD4^+^IL17A^+^ cells and CD4^+^IL17A^+^TNF-α^+^ cells. (**B**) Percentage of hepatic CD8^+^ IL17A^+^ (Tc17) cells. (**C**) Levels of IL-17A production from hepatic leukocytes after 24-hour in vitro stimulation with anti-CD3/anti-CD28 antibodies, measured by ELISA. (**D**) Quantification of CD4^+^IFNγ^+^, CD4^+^IFNγ^+^TNF-α^+^ and CD4^+^TNF-α^+^ subsets, (**E**) and CD4^+^FOXP3^+^IL17A^+^ and CD4^+^FOXP3^+^ IFNγ^+^ cells, measured by flow cytometry. (**F**) Hepatic CD68 protein levels assessed by IHC. Representative images of CD68 staining (left) and quantification plots (right). Original magnification, x200. (**G**) Representative flow cytometry plots (left) and quantification graphs of MHCII^+^ expression out F480^+^Ly6C^Mid^ and F480^+^Ly6C^hi^ cells (right). (**H**) Linear regression analysis of the association between Th17 cells and MHCII total expression out of F480^+^Ly6C^+^ cells, measured by flow cytometry. (**I**) Representative images of flow cytometry plots (left) and quantification graphs of cDC1 and cDC2 cells (right). (**J**) Linear regression analysis of the association between Th17 cells and cDC2 frecuency. Data were analyzed using the 2-tailed unpaired *t* test in **A**, **C**–**E**, **G**, and **I**. 2-way ANOVA multiple comparisons test was used in **F**. Data are displayed as mean ± SEM. (ND/Vehicle *n =* 4, HSD/Vehicle *n =* 5, ND/CCl_4_ *n =* 5, HSD/CCl_4_ *n =* 5). Data were generated in a single experiment.

Then, we characterized the immune hepatic microenvironment of the injured livers by flow cytometry. We observed that the frequency of Th17 cells (IL-17A^+^CD4^+^ T cells) was significantly higher in the injured livers of mice receiving HSD compared with ND (Figure 7A). Furthermore, the proportion of Th17 cells producing TNF-α was also elevated in the HSD group (Figure 7A), indicating that these cells may exhibit a more potent inflammatory response. Similarly, the expression of IL-17A out of CD8^+^ cells was higher in the HSD group (Figure 7B), whereas no difference was found in the production of IFN-γ and TNF-α in this population between the groups (Supplemental Figure 7A). We further assessed cytokine production by hepatic leukocytes cultured under TCR stimulation for 24 hours. Among the cytokines tested, only IL-17A was significantly increased in mice fed HSD (Figure 7C). No significant differences were observed between groups for Th1 cells (IFN-γ^+^CD4^+^), Th1 cells producing TNF-α, or the CD4^+^TNF-α^+^ subset (Figure 7D), indicating that HSD specifically promotes hepatic Th17 cell polarization without affecting other effector subsets. Interestingly, we detected an increase in proinflammatory Th17-like Tregs (IL-17A^+^ Tregs) and Th1-like Tregs in the injured livers of the HSD group (Figure 7E). These findings further underscore the role of HSD in promoting proinflammatory responses in the context of liver damage.

Also, additional characterization of the hepatic immune microenvironment revealed elevated macrophage infiltration in the injured livers of HSD-treated mice. IHC showed increased numbers of CD68^+^ cells in the livers of mice fed with HSD (Figure 7F). Consistently, flow cytometry analysis demonstrated a significant elevation in CD11b^+^F4/80^+^Ly6C^mid^-infiltrating macrophages and CD11b^+^F4/80^–^Ly6C^hi^-infiltrating monocytes in HSD-fed mice, while the frequency of resident macrophages (Ly6C^–^) remained unchanged (Supplemental Figure 7B). Notably, although the proportion of mature monocyte-derived macrophages (Mo-MΦς) (F4/80^+^Ly6C^+hi^) remained unaffected, these cells exhibited increased MHC II expression (Figure 7G). This was also observed in F4/80^+^Ly6C^mid^ macrophages, suggesting an enhanced antigen-presenting capacity of these proinflammatory macrophages under HSD conditions (Figure 7G). Consistent with the T cell results above, Th17 cell frequencies positively correlated with the percentage of MHC II expression in Mo-MΦς (Figure 7H), indicating that these macrophages may contribute to the expansion or maintenance of Th17 cells in the fibrotic liver microenvironment. Furthermore, analysis of dendritic cell subsets also revealed a substantial impact of dietary salt intake on the composition of these antigen-presenting cells. Specifically, the percentage of conventional type 2 dendritic cells (cDC2) was significantly higher in the HSD group (Figure 7I). In contrast, no significant difference was observed in the cDC1 population between groups (Figure 7I). cDC2 cells are known to play a crucial role in promoting Th17 cell differentiation through their production of key cytokines, including IL-23, IL-6, and TGF-β (26, 27). This relationship was evident in our study, as we observed a strong positive correlation between the cDC2 population and Th17 cells in the injured livers (Figure 7J). This correlation suggests that the increased presence of cDC2 cells in the HSD group may also contribute to the enhanced Th17 response observed under these conditions.

### Sodium-induced hepatocyte IL-6 production promotes Th17 cell differentiation via NF-B activation.

**Figure 8:**
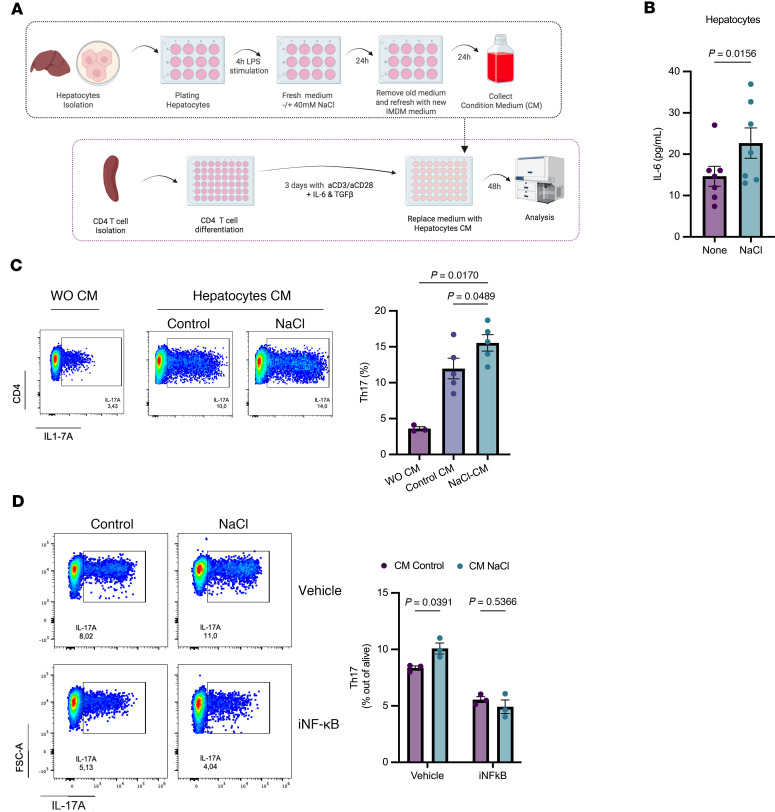
NaCl-induced hepatocyte IL-6 production promotes Th17 cell differentiation via NF-κB activation. (**A**) Schematic representation of the experimental design. Primary mouse hepatocytes were prestimulated with LPS and then cultured with or without 40 mM NaCl for 24 hours. After this period, the culture medium was removed, hepatocytes were washed, and fresh new medium (without NaCl) was added. Following an additional 24 hours, supernatants were collected and used as conditioned media (CM). (**B**) IL-6 levels in the supernatant of primary hepatocytes (*n =* 5) after LPS prestimulation and 24-hour treatment with 40 mM NaCl. (**C**) IL-17A production by CD4^+^ cells prestimulated for 3 days under Th17 differentiation conditions, then cultured for 2 additional days with normal T cell medium or with CM from hepatocytes cultured under normal (Ctrl) or high NaCl conditions. Representative flow cytometry plots (left) and quantification graphs of IL17A^+^ cells (right) (*n =* 5). (**D**) The experiment in **C** was repeated with the addition of the NF-κB inhibitor HY-13982 (10 mM) to the hepatocyte culture. Representative flow cytometry plots (left) and quantification graphs of CD4^+^IL17A^+^ T cells under each condition (*n =* 3). Data are presented as mean ± SEM and analyzed using Wilcoxon matched-pairs test for **B**, ANOVA Holm-Šídák’s multiple comparison for **C** and 2-way ANOVA Šídák’s multiple comparison for **D**. Data are representative of 2 independent experiments (*n* = 2–4 per group).

To investigate the mechanisms underlying elevated sodium-induced Th17 differentiation within the hepatic microenvironment, we analyzed cytokine production by primary hepatocytes. Hepatocytes were isolated from untreated mice, primed with LPS, and then cultured in medium supplemented with 40 mM NaCl or control medium for 24 hours (Figure 8A). Of all the cytokines assessed, only IL-6 exhibited increased production by hepatocytes cultured under high-salt conditions (Figure 8B). Furthermore, conditioned medium (CM) from hepatocytes was used to culture CD4^+^ T cells that had been prestimulated under Th17 differentiation conditions for 3 days (Figure 8A). We found that CM from control hepatocytes promoted Th17 cell differentiation, which was even further increased when the hepatocytes were previously treated with NaCl (Figure 8C). Given that NF-κB is a key transcription factor that directly regulates *Il-6* gene expression (28), we examined whether this effect was mediated by NF-κB. Importantly, NF-κB inhibition abrogated the increase in Th17 differentiation induced by CM from both control hepatocytes and those previously exposed to high NaCl conditions (Figure 8D).

Next, given that high salt intake in the CCl_4_ model resulted in increased liver fibrosis in vivo, we asked whether CM from hepatocytes in a high-salt environment could activate hepatic stellate cells (HSCs). For this, primary HSCs were isolated as previously described (29) and cultured for 48 hours with the CM from hepatocytes exposed to high NaCl. However, we did not observe a significant difference in the transcriptional expression of HSC activation markers under these conditions (Supplemental Figure 8A). Overall, these findings suggest that elevated sodium levels exacerbate liver damage and promote Th17-associated responses during profibrotic liver injury in mice. Mechanistically, increasing sodium concentration may lead to NF-κB activation and IL-6 production from hepatocytes, which, in turn, may contribute to the differentiation of hepatic Th17 cells.

## Discussion

Our study investigated the impact of dietary salt intake on CD4^+^ T cell responses and clinical parameters in patients with compensated cirrhosis. We found that high salt intake promotes Th17-driven inflammation, which is associated with a phenotype of impaired liver function. Analysis of RNA-seq data confirmed the presence of salt-induced and pathogenic Th17 signatures in liver and peripheral CD4^+^ samples of patients with liver disease. Notably, a 3-month dietary intervention aimed to reduce salt intake substantially lowered T cell-mediated inflammation, including Th17, Th1, and Th17-Th1 cells. Notably, the reduction in Th1 cells occurred even though their frequencies at baseline did not correlate with salt intake, suggesting that moderate salt restriction could initiate broader immunomodulatory effects. In fact, these effects might be the consequence of mitigating the Th17-driven proinflammatory environment, which, in turn, may contribute to dampen other proinflammatory T cell pathways. In summary, these findings suggest that moderate salt restriction may help manage immune dysregulation in individuals with compensated cirrhosis. In mice, we found that HSD exacerbated CCl_4_-induced liver fibrosis and skews immune responses towards a Th17-dominant profile. HSD increases IL-17A production and is associated with increased recruitment of inflammatory macrophages and dendritic cells. These findings suggest that HSD may indirectly promote Th17 cell differentiation by enhancing antigen presentation to T cells and creating a microenvironment that favors Th17 differentiation. In addition, our data suggests that NF-kB activation and IL-6 production from hepatocytes exposed to high sodium may also contribute to Th17 differentiation in this setting.

Our results align with current literature showing that sodium can modulate immune cell differentiation and function (13), particularly in Th17 cells (15, 16). Furthermore, research has suggested that HSD can alter the composition of the microbiota, leading to a reduction in *Lactobacillus murinus* and the promotion of proinflammatory Th17 cells in mice (21). Human Treg cells under high salt conditions have impaired suppressive function and shift towards a Th1-like profile (17–19). HSD also increases circulating monocytes in mice and humans,and can induce proinflammatory human monocytes (30–32). Additionally, sodium promotes human M1 macrophages while suppressing M2 macrophages, which may have an indirect effect on Th17 cells through the increase in proinflammatory cytokines like IL-6 and IL-1β (33, 34). Moreover, HSD can modulate the activity of myeloid-derived suppressor cells (MDSCs), inhibiting tumor growth in mice (35, 36).

The relatively small size of our patient cohort represents a limitation of our study, particularly given the potential for reporting error and bias of variables such as salt intake. We implemented different strategies to minimize this error, including the cross validation with urinary sodium excretion, which is an objective means to analyze sodium intake. Nonetheless, further studies with larger cohorts will be essential to validate our findings. Similarly, the short duration of our nutritional intervention may have limited our ability to detect changes in liver function markers, although we observed notable improvements in inflammatory parameters. The latter suggests that even a short period of salt restriction may still offer beneficial effects by modulating immune function. Future research with extended follow-up periods could provide more comprehensive insights into the long-term effects of salt restriction on both liver function and overall health outcomes in this population. Also, individual responses to dietary interventions may vary, highlighting the potential benefit of personalized approaches to salt restriction, as we implemented. Furthermore, the scope of our study did not allow for an extensive investigation of the underlying mechanisms linking salt intake with Th17 cell activation and liver function in patients with compensated cirrhosis, although our in vitro data suggest that NF-B activation and IL-6 production in hepatocytes might be a contributing factor. The mechanism whereby sodium promotes Th17 cell differentiation intrinsically in T cells has already been described (15, 16). Further mechanistic studies are needed to verify whether the same pathway is active in T cells from these patients. Similarly, future studies may also explore how changes in salt intake might influence the gut microbiota, which in turn may affect immune responses and liver function. Last, our findings are mainly concerned with systemic immunity. Systemic immune profiles derived from PBMCs do not necessarily mirror the intrahepatic immune microenvironment and therefore do not allow for conclusive statements regarding liver-specific immune responses.

In our clustering analysis, although statistically significant differences were observed between clusters, the liver function parameters remained mostly within normal clinical ranges. This likely reflects the homogeneity of our cohort, as all patients were Child-Pugh A and therefore had preserved liver function. However, it is noteworthy that several patients within cluster 2 exhibited bilirubin, AST, ALT and platelets levels outside our hospital’s reference clinical values, which was not observed in cluster 1. In addition, patients with higher dietary salt intake (cluster 2) were younger. Intriguingly, recent findings indicate that older adults have increased production of Th17-related cytokines, such as IL-17A and IL-17F, due to higher localization of STAT3 in aging T cells (37). Similarly, increased Th17 differentiation has been observed in aged mice (38). These results suggest that the heightened Th17 profile in patients with high salt consumption is not attributable to younger age, but rather underscores the role of salt intake as a Th17-inducing factor.

Physiological alterations associated with cirrhosis, such as changes in sodium and fluid homeostasis or hormonal regulations, may lead to an increased preference for salty foods, raising the question of whether high salt intake is a consequence of the disease instead of a contributing factor to its progression. Although our current data do not allow us to conclusively establish causality, it does not support this hypothesis. Patients in our cohort were not hyponatremic, and we found no evidence of dysregulated sodium balance. Renal function was preserved, and liver function was similar across groups, since all patients were Child-Pugh A. Together, this argues against a physiologic tendency to consume more sodium in our cohort. In addition, our data establish a clear connection between salt intake and systemic immune dysregulation in these patients, which is known to affect cirrhosis development.

Salt intake is associated with hypertension, cardiovascular disease, metabolic syndrome, obesity, and MASLD (39–41). Although the WHO recommends a daily salt intake below 5 g/d to reduce the risk of cardiovascular disease, recent data from 53 WHO European Region member states found that 98% of these countries exceed this recommendation, with 24 nations averaging over 10 g/day (42). Patients with decompensated cirrhosis are urged to limit their sodium consumption, mainly due to the presence of ascites. However, there is a lack of consistency across clinical guidelines regarding salt restriction in patients with compensated cirrhosis, since long-term severe salt restriction may cause notable adverse events including hyponatremia or reduced calorie intake leading to malnutrition (43). Our data, however, suggests that a salt-driven exacerbation of Th17 responses may also have detrimental effects, as inflammation is crucial in the progression of cirrhosis and related complications (44, 45). Therefore, bringing down salt intake to the recommended levels (5 g/day) with personalized, well-balanced, and healthy diets that avoid severe restriction might be a good approach.

Overall, our study provides what we believe to be novel insights into the relationship between salt intake, immune modulation, and liver function markers in patients with compensated liver disease. The findings support the notion that personalized approaches to moderate salt intake can be an effective nonpharmacological strategy to reduce inflammation in this patient population, potentially slowing disease progression and improving patient outcomes over the long term.

## Methods

### Sex as a biological variable.

Both male and female patients were included in the clinical study. In the animal models, only male mice were used due to the greater resistance of female mice to disease induction in these models. However, the underlying pathogenic mechanisms targeted in these models are conserved in female mice, and thus the findings are expected to be relevant to both sexes.

### Patients and study protocol.

We performed a nondrug, open-label, nonrandomized, experimental study in patients with compensated cirrhosis at the Liver Unit of the Hospital General Universitario Dr. Balmis (HGUDB) of Alicante, Spain. Cirrhosis was diagnosed by the histology or by clinical, laboratory, and/or ultrasonographic findings. Initially, 37 patients agreed to participate in the study, from which 8 patients refused to adhere to the dietary recommendations or did not show up for the nutritional interview and were therefore excluded. Exclusion criteria were cardiovascular disease, presence of ascites, edema, treatment with diuretics, and hyponatremia. All enrolled patients self identified as white. During their first visit (visit 1), blood and 24-hour (h) urine were collected. At that time, each patient was also interviewed by a certified nutritionist, who calculated their salt intake and offered personalized guidelines to decrease salt consumption without affecting the balance of other essential nutrients or total calorie intake. These recommendations considered the individual’s lifestyle, dietary preferences, and medical conditions to enhance adherence. Six weeks later, follow up calls were performed to monitor progress and provide additional information and recommendations as needed. Participants were also given the contact information of the nutritionist and the principal investigator and were instructed to contact them with any questions or concerns. Visit 2 was scheduled for 3 months after visit 1, during which patients were interviewed again, and blood and 24h urine samples were collected again. Data on clinicopathological characteristics and hematological laboratory tests were recorded from each patient.

### Animals.

Six- to eight-week-old male C57BL/6J mice were used for all experiments. Mice were originally purchased from Jackson Laboratories (Bar Harbor, ME), and then bred under specific pathogen free (SPF) conditions in filter top cages at our animal facility at the University Miguel Hernández (UMH). For the acute liver damage model, mice were fed either a normal diet (ND, 0.6% NaCl) or a high-salt diet (HSD, 4% NaCl) in addition of 1% of NaCl in the drinking water for 3 weeks and then injected intraperitoneally (i.p.) with a single dose of 100 mg/kg diethylnitrosamine (DEN) or vehicle solution as a control. Mice were sacrificed 48 hours after the DEN injection. For the liver fibrosis model, mice were prefed with ND or HSD for one week (1% of NaCl in the drinking water). While continuing the same diet for an additional 3 weeks, they received 2 injections per week (2 mL/Kg, i.p.) of carbon tetrachloride (CCl_4_).

### Salt intake calculation.

Individual interviews were conducted to assess each participant’s sodium intake. Patients completed 24h food diaries, where they recorded what they ate and drank, along with the quantities. Sodium intake was calculated using the Evalfinut software (https://www.finut.org/evalfinut/), which utilizes the BEDCA and USDA databases. For the initial calculation of salt intake, food records from the 3 days prior to the nutritional intervention were used. For the final calculation, a 24h food record was randomly selected each week during the 3-month study period.

### Isolation of PBMCs.

Venous whole blood was collected in K3-EDTA (BD Bioesciences) tubes. PBMCs were isolated using Ficoll density gradient centrifugation (Biochrom). PBMCs at the interface were collected, washed with PBS, and counted. CD4^+^ T cells were isolated from PBMCs by immunomagnetic selection using the EasySep Human CD4^+^ T cell isolation kit (StemCell Technologies).

### Human CD4^+^ T cell isolation, culture and stimulation.

Complete RPMI 1640 RPMI + 10% FCS +2 mM L-glutamine + 1% PenStrep (Thermo Fisher Scientific) was used throughout the experiments. CD4^+^ T cells were isolated by immunomagnetic negative selection using an EasySep Human CD4^+^ T cell isolation kit (StemCell Technologies). Purity was higher than 90% in all experiments, as confirmed by flow cytometry. After enrichment, a portion of the cells was cultured in complete RPMI medium and stimulated with 5 μg/mL plate bound anti-CD3 (Invitrogen) and 1 μg/mL soluble anti-CD28 (Invitrogen) antibodies (Supplemental Table 2). Twenty-four–hour culture supernatants were collected for cytokine analysis using the Ready-Set-Go ELISA kits (Invitrogen). Another part of the cells was stimulated for 5 hours at 37^o^C and half with phorbol 12-myristate 13-acetate (PMA)/ionomycin Cell Stimulation Cocktail with Golgi Plug (BD Biosciences) for cytokine production by flow cytometry.

### Murine T cell cultures.

For Th17 cell differentiation, splenic naive CD4 T cells where isolated from 10-week-old mice by immunomagnetic negative selection of EasySep naive CD4^+^ T cell isolation kit (StemCell Technologies). The cells were stimulated by plate-bound anti-CD3 and anti-CD28 (Invitrogen) in the presence of 40 ng/mL IL-6 (PeproTech) and 1 ng/mL TGFb (PeproTech) for 3 days. After, the medium was changed for condition medium derived from hepatocytes and the cells were cultured for 2 more days.

### Hepatocyte isolation and stimulation.

For isolation of primary hepatocytes, mouse livers were perfused via the portal vein with Liver Perfusion Medium (Thermo Fisher Scientific) followed by Liver Digestion Medium (Thermo Fisher Scientific). The digested liver was dissociated in cold Williams’ E medium (Thermo Fisher Scientific) and filtered through 100 μm and 70 μm cell strainers. Hepatocytes were purified by centrifugation at 40*g* for 2 minutes, followed by density gradient centrifugation using 40% Percoll (Cytiva) solution at 200*g* for 10 minutes. Viable hepatocytes were washed, counted, and plated at 0.5 x 10^6^ cells/mL in supplemented Williams’ E medium. After 4 hours of attachment, the medium was replaced with serum-free conditions for subsequent experiments. The next day, hepatocytes were stimulated with 1 ng/mL LPS (InvivoGen) for 4 hours. Following this, the LPS was removed, and the cells were washed. Stimulation with 40 mM NaCl or without NaCl was then performed for 24 hours. In one experiment, a 10 mM NF-κB inhibitor (MedChemExpress) was added during the NaCl stimulation. After 24 hours, the medium was removed and replaced with fresh IMDM (Thermo Fisher Scientific) cell culture medium. Following another 24 hours, the medium was collected and used as CM for T cell cultures. All procedures were approved by the IACUC.

### Flow cytometry analysis on human PBMCs and CD4.

PBMCs were isolated and washed with FACs buffer (PBS containing 2% FBS and 2 mM). The pellet was resuspended in a mixture of FACs Buffer with surface antibodies (CD3, CD4, CCR10, CCR6) and incubated for 20 minutes at 4°C in the dark. Cells were then washed and acquired using BD FACSCanto.

CD4 cells, after 5-hour in vitro stimulation with PMA/ionomycin, were washed with FACS buffer, resuspended in a master mix of FACs Buffer with surface antibodies (CD3, CD4) and fixable viability dye, and incubated for 20 minutes at 4°C in the dark. Cells were washed again with FACs buffer, and intracellular staining for cytokines (IL-17A and IFN-γ) was performed according to the manufacturer’s instructions. After staining, cells were washed and acquired using BD FACSCanto. All antibodies are included in Supplemental Table 2.

Fluorescence minus one (FMO) controls, unstained controls, and single-staining controls were performed to ensure proper gating of positive populations. Data were analyzed using FlowJo software. All analyses were conducted following the guidelines for the use of flow cytometry in immunology.

### Hepatic Stallate cell isolation.

Hepatic stellate cells (HSCs) were isolated from C57BL/6J mice, as previously described (29). In brief, mice were euthanized, and livers were perfused in situ via the inferior vena cava with 30 mL of prewarmed EGTA solution. This was followed by perfusion with pronase (14 mg per mouse) and subsequently with collagenase D (3.7 U per mouse) to enzymatically digest the extracellular matrix. Postperfusion, the liver was excised, minced, and subjected to further digestion in a pronase/collagenase/DNase I solution at 37°C for 25 minutes. The resulting suspension was filtered through a 70 μm cell strainer, washed twice with Gey’s balanced salt solution B (GBSS/B). Then the cells were resuspended in GBSS/B and mixed with 11.5% HistoDenz (D2158 Sigma-Aldrich) solution. The suspensions were overlaid carefully with 1.5 mL GBSS/B (Sigma-Aldrich) and centrifuged at 1,380*g* for 17 minutes at 4°C. Cells located at the interface of the HistoDenz solution and the GBSS/B were harvested and collected in a 50 mL Falcon tube and washed once with GBSS/B. The isolated HSCs were collected, counted, and cultured in DMEM with 10% FBS for 48 hours for activation studies.

### Flow cytometry on murine hepatic leucocytes.

Isolation of hepatic leukocytes was performed as described before (46). Briefly, livers were perfused in situ and subsequently harvested, then immediately placed in cold PBS (Invitrogen). The liver tissue was mechanically dissociated by pressing it through a 70 μm cell strainer using the plunger of a 3 mL syringe in cold PBS with PBS to obtain a single-cell suspension. Cells were pelleted by centrifugation at 500*g* for 5 minutes at 4°C. The pellet was resuspended in 5 mL of 40% isotonic Percoll (Cytiva) and gently overlaid onto 3 mL of 60% isotonic Percoll in a 15 mL conical tube. Gradient centrifugation was performed at 800*g* for 20 minutes at 4°C with acceleration set to 5 and deceleration to 0 (no brake). The upper layer containing fat and debris was discarded, and lymphocytes were collected from the interphase. The cells were washed using FACs buffer. Up to 1 × 10^6^ lymphocytes were transferred into flow cytometry tubes, pelleted at 400*g* for 5 minutes at 4°C, and subsequently resuspended in 50 μL of a staining mixture with CD11b, F480, Ly6C, Ly6g, CD11c, MHCII, and a viability dye. Following a 30-minute incubation at 4°C, the cells were washed with FACs Buffer and then acquired using the BD FACSCanto cytometer.

For T cell characterization, following Percoll separation, cells were stimulated with PMA/ION for 4 hours. Subsequently, cells were washed with FACS buffer, resuspended in surface master mix antibodies (CD3, CD4, CD8, and a viability dye), and incubated for 20 minutes at 4°C in the dark. Intracellular staining was performed using the eBioscience Foxp3/Transcription Factor Staining Buffer Set (Invitrogen) in accordance with the manufacturer’s instructions. The cells were then resuspended in a staining master mix containing IL-17A, IFNγ, TNF-α, and FOXP3 and incubated overnight at 4°C in the dark. The next day, cells were washed with FACs Buffer and then acquired using the BD FACSLyric cytometer. Detailed information on all antibodies used is provided in Supplemental Table 2.

### Cytokine quantification.

ELISA for the quantitative measurement of cytokine production were performed using Ready-SET-Go ELISA kits (Thermo Fisher Scientific), according to the manufacturer’s instructions. All samples were tested in duplicates. Standard curves were generated for each plate and the average optical density of the zero standard was subtracted from the rest of the standards and samples to obtain a corrected concentration for all cytokines.

### Histology and IHC.

Mouse fresh liver tissues were harvested, 10% formalin-fixed, paraffin-embedded, sliced into 5-μm sections and subjected to standard H&E staining and immunostaining. The detection of IL-17A, α-SMA, and CD68 by IHC was carried out via deparaffinization and rehydration of the tissue section with xylene and decreasing strengths of alcohols (100%, 96%, and 70% ethanol) followed by water. Antigen retrieval was performed by heating in a microwave with a solution of sodium citrate 1 M, pH 6. Blocking of the endogenous peroxidase, the nonspecific binding of the antibody and the nonspecific endogenous signal from avidin biotin was performed following standard protocols. The primary antibody was incubated (anti-IL-17A, CD68, or ACTA2) followed by incubation with a secondary antibody, specifically a biotinylated goat anti-Rat IgG. To amplify the signal, the peroxidase Avidin–Biotin Complex kit was used (Vectastain Peroxidase Standard ABC kit) and the signal was detected with diaminobenzidine (DAB) peroxidase substrate kit (Vector Laboratories), followed by dehydration of the tissue and mounting with Eukitt. For necrosis quantification, 3–4 low magnification images (10X) of the most severely damaged areas of each liver were taken, and then the percentage of necrotic area in each image was calculated using ImageJ software (http://imagej.nih.gov/ij/).

### Isolation of RNA and quantitative real-time PCR.

RNA isolation was performed using a RNeasy Mini Kit (Qiagen) according to the manufacturer’s instructions. Then, to avoid unwanted DNA amplification, all samples were pretreated with a DNA-free DNA removal kit (Thermo Fisher Scientific). For gene expression analysis, 10 ng of RNA sample was then used for one-step qPCR using a qScript One-Step RT-qPCR kit (Quantabio). GAPDH expression was used as an internal reference in all experiments, and all samples were tested in duplicate. The qRT-PCR primers for each specific target gene were designed based on their reported sequence. The calculation of mRNA fold induction was performed using the double delta Ct (cycle threshold) method. The average delta Ct of the control group in each experiment was used as the reference value to calculate the double delta Ct for each individual sample in each group.

### Reanalysis of published transcriptomic datasets.

RNA-seq data were downloaded from the Database of Genotypes and Phenotypes (dbGAP) of the National Center for Biotechnology Information under accession number phs001807.v1.p1 and the Gene Expression Omnibus with GEO accession code GSE102005. Differential expression between groups was assessed using moderated *t* statistics. Enrichment analyses of gene signatures associated with pathogenic Th17 (25) and sodium-treated Th17 (15) cells were performed using the GSEA function in the gene set enrichment package (gseGO) in R, based on the previously computed *t* statistic values. Heatmaps were constructed using R statistical software. The list of genes from the pathogenic and sodium-treated Th17 signatures is provided in a separate supporting file.

### Statistics.

Quantitative variables were expressed as median and interquartile range or mean and standard error, and categorical data were expressed as frequency (percentages). Mann-Whitney U test was used to compare quantitative variables between 2 groups, while categorical variables were compared using chi-squared or Fisher’s exact test as appropriate. Correlations between quantitative variables were analyzed using the Spearman test.

Unsupervised hierarchical clustering analysis was performed to classify patients into different clinical subgroups by the Ward’s minimum variance linkage method using the squared Euclidean distance metric. Quantitative variables included in the clustering analysis are detailed on Table 1.

Statistical analyses were performed using GraphPad Prism version 9.0.0 (GraphPad software) and R version 4.3.0 (R Core Team). Two-tailed significance test with a *P* value 0.05 was considered significant.

### Study approval.

Written informed consent was obtained from each participant, the protocol was supervised and approved by the Clinical Research Ethics Committee (CEIm) of our hospital (approval number PI2019/114), and all research was conducted in accordance with both the Declarations of Helsinki and Istanbul.

For mice studied, all experimental procedures were approved by the Office of Agriculture, Rural Development, and Climate Change of the Generalitat Valenciana (approval number 2020/VSC/PEA/0100), ensuring that all animals received humane care in accordance with Spanish and European legislation.

### Data availability.

The RNA sequencing datasets analyzed are available at the Database of Genotypes and Phenotypes (dbGAP) of the National Center for Biotechnology Information under accession number phs001807.v1.p1 and the Gene Expression Omnibus with GEO accession code GSE102005. The code used and the results from the analysis of these datasets is available at https://github.com/Juanjo-Bioinfo/Tzoumpa2025; commit ID 7665da5 and b30ec33. The rest of the data used or analyzed during the current study are available from the corresponding author upon reasonable request. Values for all data points in graphs are reported in the Supporting Data Values file.

## Author contributions

JMGN designed the research studies and acquire funding. AT and BLR conducted the experiments and acquire data. AT, BLR, YH, and JP, contributed to the in vivo mouse models. AM performed the nutritional evaluation and follow up of patients. MTP, IH, MR, CM, PB, and SP enrolling and clinical follow up of patients. PP and FT contributed to the flow cytometry experiments. JL performed the bioinformatic analyses. JMGN and AT conducted data analyses. JMGN and AT wrote the initial manuscript, and PZ and SP reviewed manuscript. PZ helped with data analysis and was consulting statistician. All authors have read and agreed to the published version of the manuscript.

## Supplementary Material

Supplemental data

ICMJE disclosure forms

Supplemental data set 1

Supporting data values

## Figures and Tables

**Table 1:**
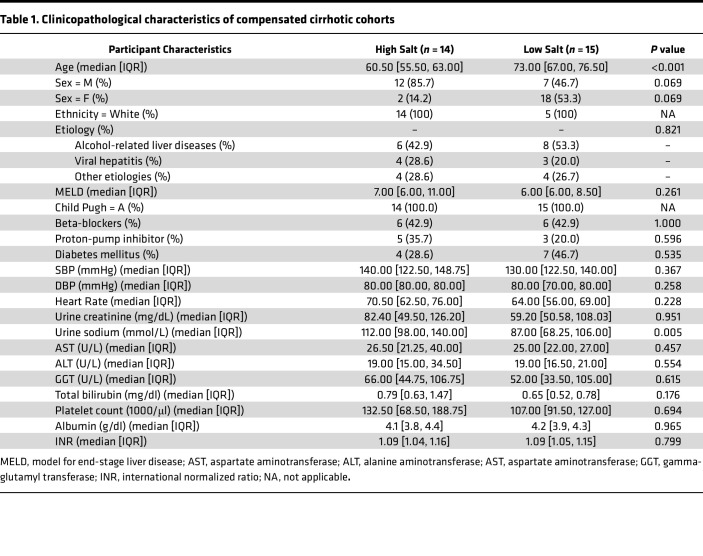
Clinicopathological characteristics of compensated cirrhotic cohorts

## Version 1 07/24/2025

In-Press Preview

## Version 2 09/09/2025

Electronic publication
