# Supplementary material for: Dietary salt intake worsens the Th17-dependent inflammatory profile of patients with cirrhosis
Source: JCI Insight. 2025 Jul 24;10(17):e191354. doi: 10.1172/jci.insight.191354 (PMC12487690; doi:10.1172/jci.insight.191354)
Supplement: Supplemental data [file jciinsight-10-191354-s193.pdf]

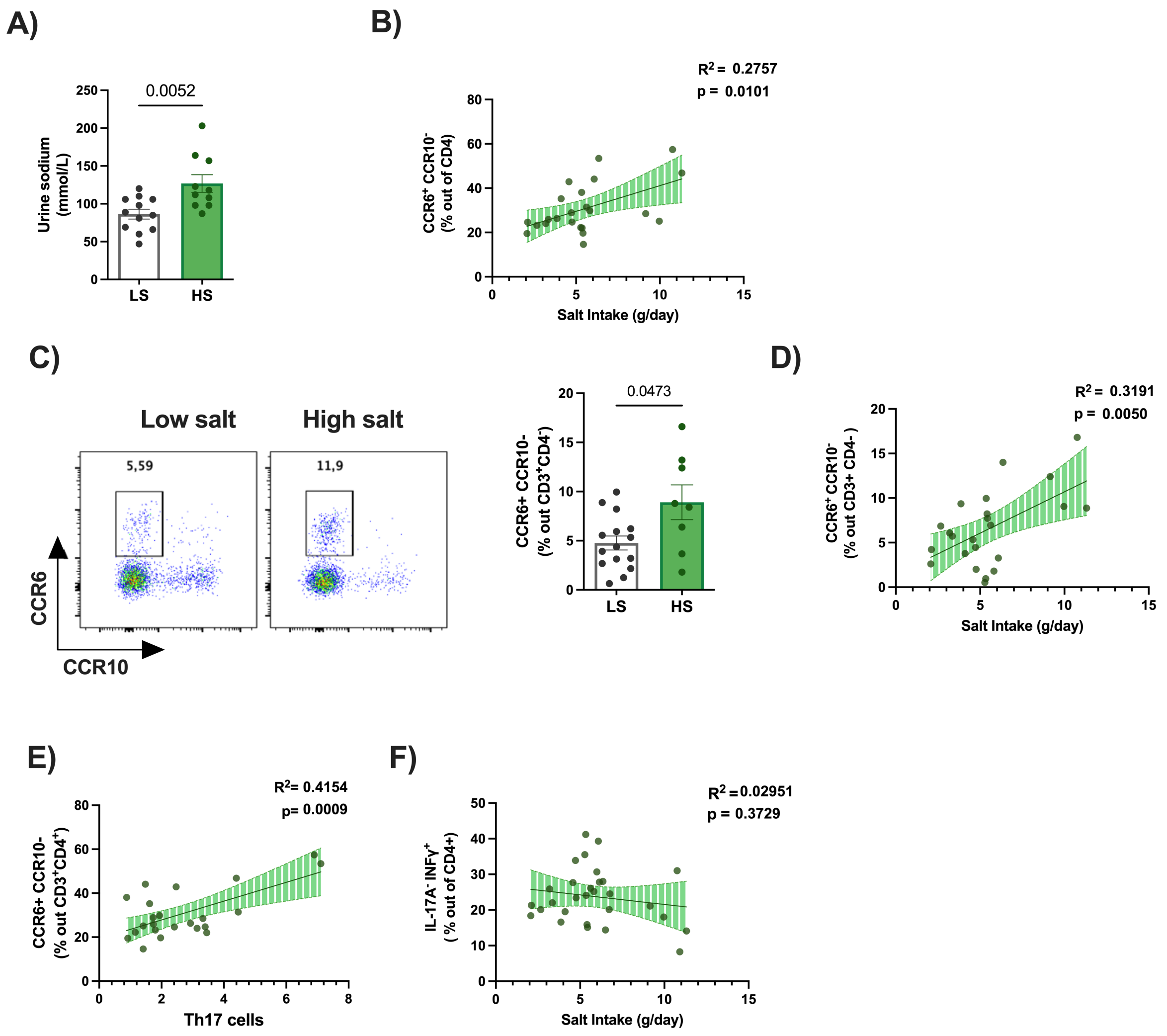

Supplemental Figure 1: Association between dietary salt intake and circulating T cell subsets

(A) Quantification of 24-hour urinary sodium excretion in patients on high-salt (HS, n=10) and low-salt (LS, n=12) diets. **(B)** Linear regression analysis showing the association between daily salt intake and the percentage of circulating $CD4^{+}CCR6^{+}CCR10^{-}$ (n=23). **(C)** Representative flow cytometry plot (left) and quantification (right) of $CCR6^{+}CCR10^{-}$ cells in $CD3^{+}CD4^{-}$ population from both groups of patients (n=8/HS and n = 15/LS). **(D)** Linear regression analysis of the association between daily salt intake and the frequency of circulating $CCR6^{+}CCR10^{-}$ (out of $CD3^{+}CD4^{-}$) (n=23). **(E)** Linear regression analysis of the association between $CD4^{+}CCR6^{+}CCR10^{-}$ cells and circulating Th17 ($CD4^{+}IL\text{-}17A^{+}$) cells (n=23). **(F)** Linear regression analysis of the association between daily salt intake and the presence of Th1 cells ($CD4^{+}IL17A^{-}IFN\gamma^{+}$) (n=29). Quantitative data in A) and C) were analyzed using the Mann-Whitney U test and are displayed as mean ± SEM.

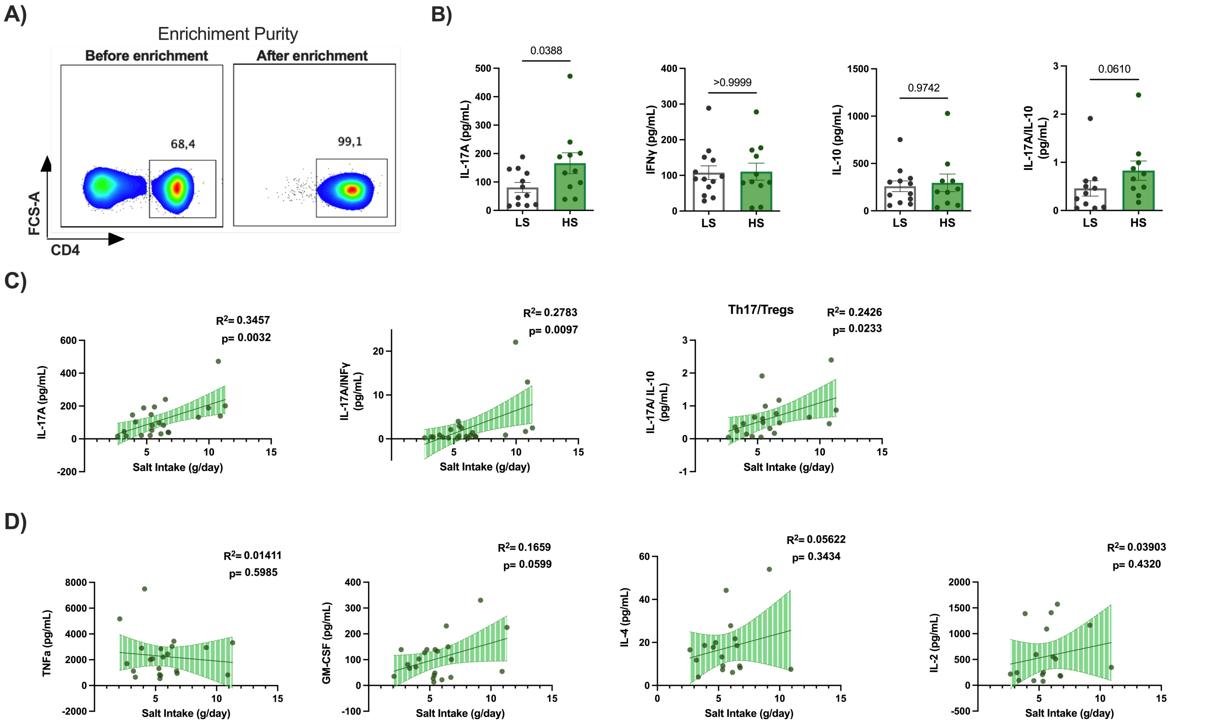

Supplemental Figure 2: Analysis of cytokine production in supernatants from in vitro stimulated CD4⁺ T cells.

CD4⁺ T cells were isolated from PBMCs by magnetic enrichment and stimulated in vitro with anti-CD3 and anti-CD28 antibodies for 24 hours. **(A)** Representative flow cytometry plot of CD4⁺ cells before and after isolation, showing 99% purity after magnetic enrichment. **(B)** Quantification plots display protein levels of IL-17A, IFNγ, IL-10, and the IL-17A to IL-10 ratio in the supernatant of in vitro stimulated CD4⁺ T cells from patients on high-salt (HS, n=11) or low-salt (LS, n=12) diets. **(C)** Linear regression analysis showing the association between daily salt intake and the production of IL-17A, the ratio of IL1-7A to IFNγ, and the ratio of IL1-7A to IL-10 (n=23). **(D)** Linear regression analysis of daily salt intake and the levels of TNFα, GM-CSF, IL-4 and IL-2 from the supernatants of CD4⁺ T cells stimulated in vitro for 24 hours. Data in B) was analyzed using the Mann-Whitney U test and are represented as mean ± SEM.

A)

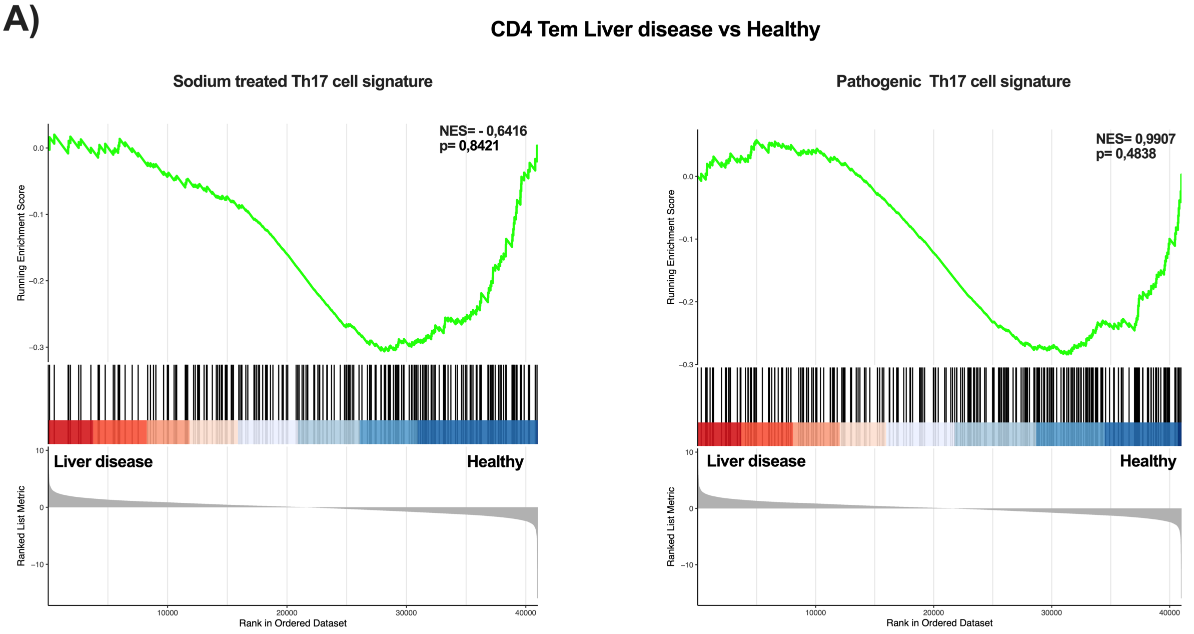

Supplemental Figure 3: Sodium-associated gene signatures in effector memory CD4⁺ T cells from patients with liver disease

(A) Gene Set Enrichment Analysis (GSEA) plots showing enrichment of sodium-induced Th17 gene signature (left) and pathogenic Th17 signature (right) in bulk RNA-seq data from effector memory CD4 T cells derived from peripheral blood of patients with liver disease (n=9) or healthy individuals (n=8)

A)

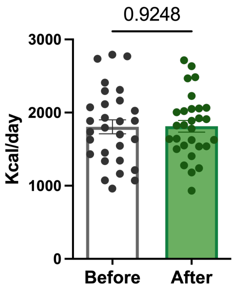

B)

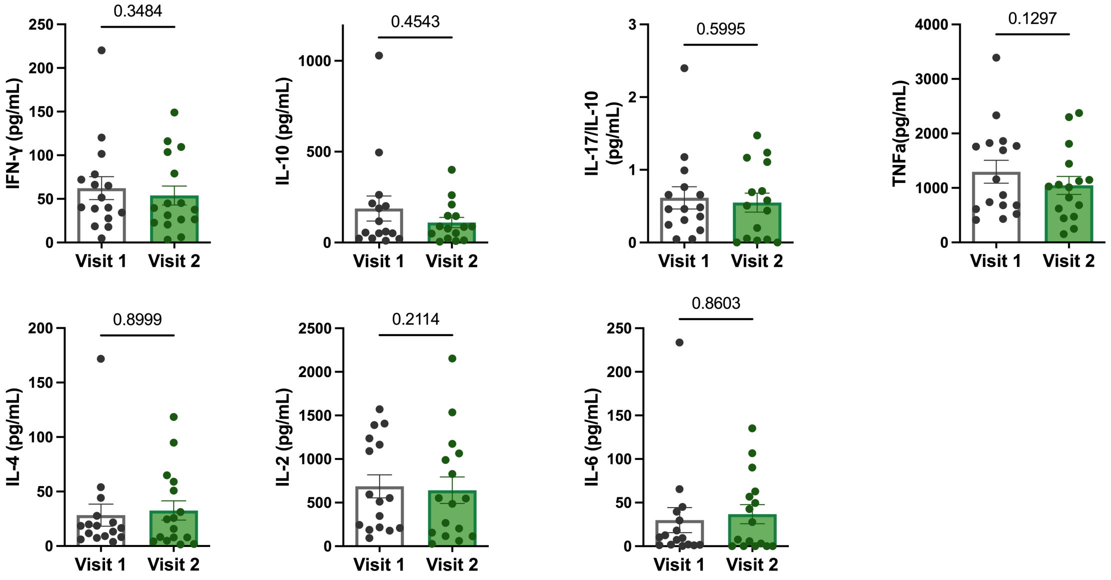

Supplemental Figure 4: Effect of salt restriction on cytokine production by peripheral CD4$^+$ T cells in vitro

(A) Total calories intake for each patient before and after the salt restriction protocol.**(B)** Paired analysis of IFNγ, IL-10, TNFα, IL-6, IL-2, and IL-4 production by peripheral CD4$^+$ T cells cultured in vitro for 24h, along with the IL-17 to IL-10 ratio, in cirrhotic patients before and after salt restriction (n=25). Data were analyzed using Wilcoxon matched-pairs test and are displayed as mean ± SEM.

A)

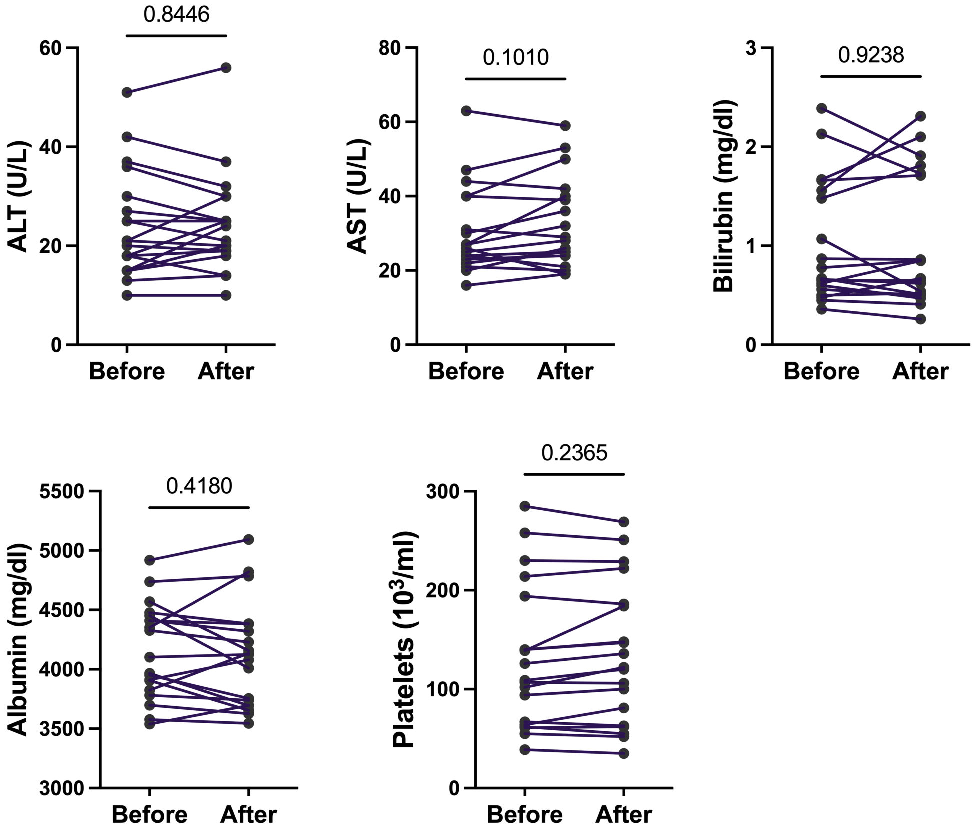

Supplemental Figure 5: Effect of salt restriction on quantitative clinical variables

(A) Paired analysis of quantitative clinical parameters of liver function in patients before and after salt restriction (n=25). Data were analyzed using Wilcoxon matched-pairs test and are displayed as mean ± SEM.

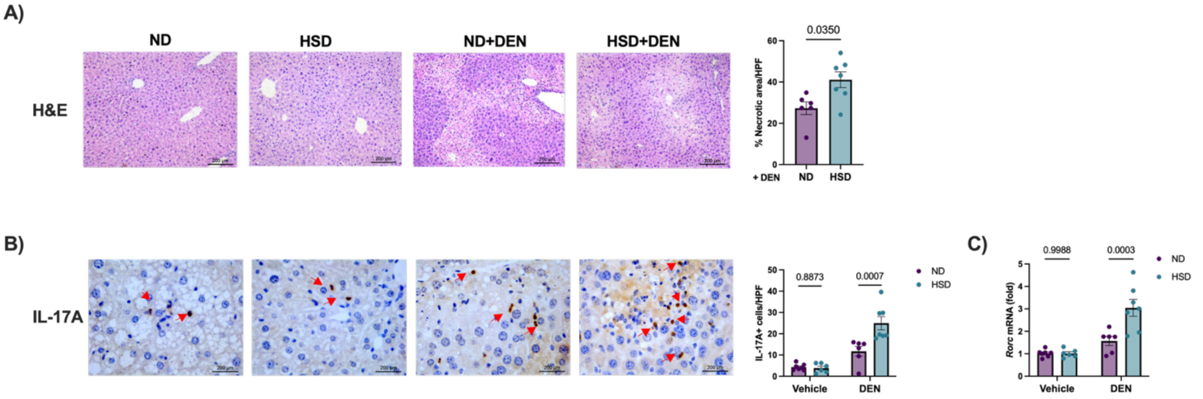

Supplemental Figure 6: High salt diet exacerbates diethylnitrosamine (DEN)-induced liver damage

Mice were fed with normal diet (ND) or high salt diet (HSD) and injected with diethylnitrosamine (DEN) or vehicle as control (n=6-7 per group). **(A)** Assessment of acute liver injury by hematoxylin and eosin (H&E) staining of liver tissue sections. Representative histological images (left) and quantitative analysis of the percentage of necrotic area in each group of mice (right) **(B)** IL-17A protein levels in liver tissue assessed by immunohistochemistry. Representative images of IL-17A staining (left) and quantification plots of IL-17A^{+} cells per field (right). **(C)** mRNA expression of the Th17-associated transcription factor *Rorc* in liver tissue. Data were analyzed using the Mann-Whitney U test (A) or ANOVA with multiple comparisons test (B and C) and are displayed as mean ± SEM.

A)

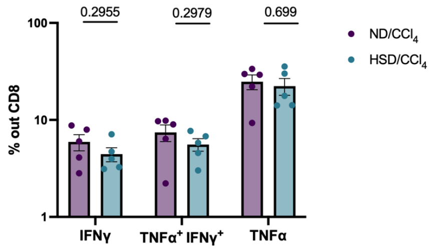

B)

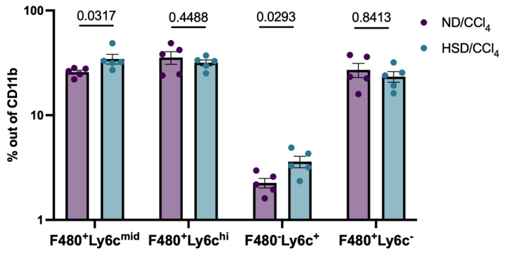

Supplemental Figure 7: Analysis of hepatic CD8+ and CD11b+ populations from mice with CCl_4-induced fibrosis

(A) Quantification of IFNγ$^+$, TNFα$^+$ and IFNγ$^+$TNFα$^+$ cells within the hepatic CD8$^+$ population from the liver of mice with CCl_4-induced fibrosis, measured by flow cytometry analysis. **(B)** Quantification plots from flow cytometry analysis of infiltrating macrophages (F4/80$^+$Ly6C^{mid}), mature monocyte-derived macrophages (F4/80$^+$Ly6C^{hi}), infiltrating monocytes (F4/80$^-$Ly6C^{hi}) and resident macrophages (F4/80$^+$Ly6C$^-$) in the liver of mice with CCl_4-induced fibrosis. (ND/Vehicle n=4, HSD/Vehicle n=5, ND/CCl_4 n=5, HSD/CCl_4 n=5). Data were analyzed using Mann-Whitney U and are displayed as mean ± SEM.

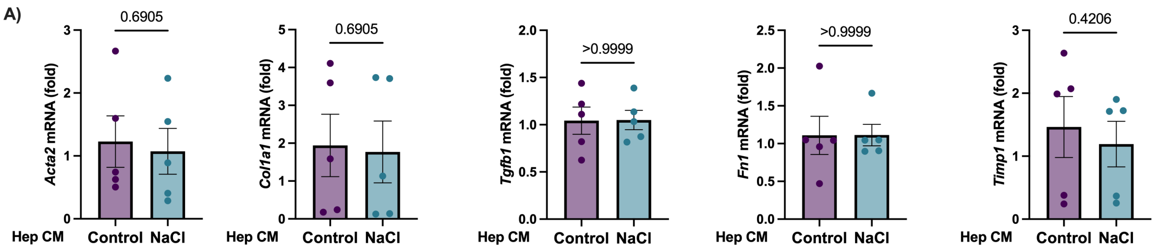

Supplemental Figure 8: Conditioned media from sodium-treated hepatocytes does not modulate hepatic stellate cell activation

Primary hepatic stellate cells (HSCs) were cultured with conditioned media (CM) from hepatocytes previously exposed to high salt (NaCl, 40mM) or control conditions (n=5 per group). **(A)** mRNA expression levels of fibrogenic markers (*Acta2, Col1a1, Tgfb1, Fn1, Timp1*) in HSCs. Data were analyzed using the Mann-Whitney U test and are displayed as mean ± SEM.

Supplemental Tables and Figures

Supplemental Table 2 Clustering Analysis

Parameters	Cluster 1 (Median [IQR])	Cluster 2 (Median [IQR])	p-value
Salt (g)	5.28 [3.87, 5.82]	9.56 [6.47, 10.80]	<0.001
Age (years)	69.00 [63.00, 75.00]	59.00 [56.25, 63.25]	0.010
MELD Score	6.00 [6.00, 8.25]	8.50 [6.00, 11.00]	0.154
SBP (mmHg) (median [IQR])	130.00 [120.00, 140.00]	142.50 [131.25, 150.00]	0.210
DBP (mmHg) (median [IQR])	80.00 [70.00, 80.00]	80.00 [80.00, 81.25]	0.148
Heart Rate (median [IQR])	64.00 [56.00, 73.00]	72.00 [63.50, 76.00]	0.220
Glucose (mg/dL)	102.00 [94.00, 130.00]	111.50 [104.50, 120.00]	0.241
Urea (mg/dL)	30.00 [26.00, 36.00]	27.00 [19.00, 35.00]	0.317
Creatinine (mg/dL)	0.81 [0.76, 0.92]	0.74 [0.71, 0.84]	0.447
Sodium (mmol/L)	140.00 [139.00, 141.00]	140.00 [139.00, 141.00]	0.900
Potassium (mmol/L)	4.30 [4.20, 4.50]	4.00 [3.80, 4.30]	0.099
Chloride (mmol/L)	103.00 [101.00, 105.00]	103.00 [101.25, 104.25]	0.640
Calcium (mg/dL)	9.30 [9.20, 9.60]	9.10 [8.93, 9.15]	0.006
Inorganic Phosphate (mg/dL)	3.45 [3.10, 3.80]	2.80 [2.70, 3.35]	0.063
Total Proteins (g/dL)	7.00 [6.70, 7.15]	7.10 [7.00, 7.20]	0.271
Uric Acid (mg/dL)	5.40 [4.60, 6.30]	5.25 [4.62, 6.73]	0.732
Cholesterol (mg/dL)	168.00 [149.00, 196.00]	167.50 [140.00, 182.50]	0.770
Triglycerides (mg/dL)	82.00 [71.00, 103.00]	95.00 [82.50, 165.75]	0.341
Alkaline Phosphatase (U/L)	95.00 [62.00, 123.00]	105.50 [91.25, 169.75]	0.172
Magnesium (mg/dL)	2.00 [1.93, 2.10]	1.83 [1.78, 1.84]	0.078
Leukocytes (×10^3/μL)	4.85 [4.06, 6.95]	5.30 [3.33, 5.94]	0.696
Neutrophils (%)	61.50 [53.60, 64.30]	63.85 [58.77, 64.25]	0.714
Lymphocytes (%)	24.70 [21.80, 32.00]	25.00 [20.75, 26.02]	0.367
Monocytes (%)	8.50 [7.30, 10.30]	9.40 [8.70, 11.43]	0.379
Erythrocytes (×10^6/μL)	4.44 [4.28, 5.15]	4.98 [4.77, 5.12]	0.262
Hemoglobin (g/dL)	14.00 [13.00, 14.90]	15.75 [15.05, 16.40]	0.003
Alpha-fetoprotein (ng/mL)	2.20 [1.80, 2.70]	3.10 [2.05, 4.25]	0.305
Direct Renin (ng/L)	16.87 [8.83, 55.66]	9.56 [5.34, 13.52]	0.106
Quick (%)	91.00 [82.75, 94.50]	83.50 [78.75, 89.75]	0.414
INR	1.06 [1.04, 1.13]	1.13 [1.08, 1.17]	0.385
Total bilirubin (mg/dl) (median [IQR])	0.66 [0.52, 0.78]	1.26 [0.81, 1.53]	0.031
AST (U/L) (median [IQR])	24.00 [21.00, 27.00]	40.00 [29.25, 44.75]	<0.001
ALT (U/L) (median [IQR])	18.00 [15.00, 21.00]	33.00 [22.50, 38.25]	0.009
GGT (U/L) (median [IQR])	48.00 [23.00, 82.00]	95.50 [70.75, 133.50]	0.007
Platelet count (1000/µl) (median [IQR])	114.00 [100.00, 214.00]	70.00 [63.50, 105.50]	0.045
Albumin (g/dl) (median [IQR])	4.35 [4.00, 4.45]	3.92[3.76, 4.05]	0.013
Th17 % (median [IQR])	1.80 [1.57, 2.47]	4.02 [3.04, 5.07]	<0.001
Th1 % cells (median [IQR])	24.10 [20.10, 27.80]	19.55 [14.33, 26.58]	0.188
Ratio Th17/Th1 (median [IQR])	0.09 [0.06, 0.14]	0.27 [0.19, 0.29]	0.001

Supplemental Table 2

Antibody	Species	Clone	Catalog Number - Manufacturer	Dilution	Application
CD3 FITC	Anti-Human	OKT3	566783-BD Biosciences	1/100	Flow Cytometry
CD4 APC-H7	Anti-Human	RPA-T4	560251-BD Biosciences	1/100	Flow Cytometry
CD196 (CCR6) PerCP-Cy™5.5	Anti-Human	11A9	561752-BD Biosciences	1/100	Flow Cytometry
CCR10 APC	Anti-Human	1B5	564771-BD Biosciences	1/100	Flow Cytometry
IFN-γ PE-Cy™7	Anti-Human	B27	560924-BD Biosciences	1/100	Flow Cytometry
IL-17A Alexa Fluor® 647	Anti-Human	N49-653	560491-BD Biosciences	1/25	Flow Cytometry
CD8a BV605	Anti-Mouse	53-6.7	563152-BD Biosciences	1/200	Flow Cytometry
CD4 BV7111	Anti-Mouse	GK1.5	563050-BD Biosciences	1/200	Flow Cytometry
Ly-6C PE-Cy™7	Anti-Mouse	AL-21	560593-BD Biosciences	1/300	Flow Cytometry
Ly-6G PerCP-Cy™5.5	Anti-Mouse	1A8	560602-BD Biosciences	1/200	Flow Cytometry
Anti-CD11b BB515	Anti-Mouse	M1/70	564454-BD Biosciences	1/100	Flow Cytometry
CD4 PE-Cy™7	Anti-Mouse	RM4-5	RM4-5-BD Biosciences	1/300	Flow Cytometry
IL-17A BV421	Anti-Mouse	TC11-18H10	563354-BD Biosciences	1/100	Flow Cytometry
IFN gamma APC	Anti-Mouse	XMG1.2	47-7311-80-Invitrogen	1/100	Flow Cytometry
FOXP3 PE	Anti-Mouse	FJK-16s	12-5773-82-Invitrogen	1/100	Flow Cytometry
MHC Class II eFluor™ 450	Anti-Mouse	M5/114.15.2	48-5321-80-Invitrogen	1/400	Flow Cytometry
CD11c APC-eFluor™ 780, eBioscience	Anti-Mouse	N418	47-0114-80-Invitrogen	1/100	Flow Cytometry
Fixable Viability Dye eFluor™ 506			65-0866-14-Invitrogen™	1/500	Flow Cytometry
TNF-α PE/Cyanine7	Anti-Mouse	MP6-XT22	506323-Biolegend	1/300	Flow Cytometry
F4/80 APC	Anti-Mouse	BM8	123115-Biolegend	1/200	Flow Cytometry
Fc Blocking	Anti-Human		422302- Biolegend	1/100	Flow Cytometry
CD16/32	Anti-Mouse	93	101301-Biolegend	1/200	Flow Cytometry
IL-17A	Anti-Mouse	Polyclonal	PA5-79470-Invitrogen	1/200	Immunohistochemistry
CD68	Anti-Mouse	RM1031	ab303565 -Abcam	1/200	Immunohistochemistry
Anti-alpha smooth muscle Actin antibody (α-SMA)	Anti-Mouse	Polyclonal	ab5694 -Abcam	1/200	Immunohistochemistry
Goat Anti-Rabbit IgG Antibody (H+L), Biotinylated	Anti-Rabbit		BA-1000-1.5	1/200	Immunohistochemistry
CD3	Anti-Human	OKT3	16-0037-81-Invitrogen™	5 µg/ml	In vitro T cell activation
CD28	Anti-Human	CD28.2	16-0289-85--Invitrogen™	1 µg/ml	In vitro T cell activation
CD3	Anti-Mouse	17A2	14-0032-Invitrogen™	2 µg/ml	In vitro T cell activation
CD28	Anti-Mouse	37.51	16-0281-86-Invitrogen™	2 µg/ml	In vitro T cell activation
